# Efficacy of Low-Voltage-Area Ablation Is Enhanced in Patients With Advanced Left Atrial Enlargement: A Subanalysis of the SUPPRESS-AF Trial

**DOI:** 10.1161/CIRCEP.125.014210

**Published:** 2025-09-26

**Authors:** Masaharu Masuda, Yasuhiro Matsuda, Hiroyuki Uematsu, Hirotaka Ooka, Satoshi Kudo, Mizuki Ochi, Toshiaki Mano, Akihiro Sunaga, Nobuaki Tanaka, Tetsuya Watanabe, Hitoshi Minamiguchi, Yasuyuki Egami, Takafumi Oka, Tomoko Minamisaka, Takashi Kanda, Masato Okada, Masato Kawasaki, Koji Tanaka, Nobuhiko Makino, Hirota Kida, Shungo Hikoso, Tomoharu Dohi, Koichi Inoue, Yohei Sotomi, Yasushi Sakata, the OCVC-SUPPRESS-AF Investigators, Takuya Tsujimura

**Affiliations:** Cardiovascular Center, Kansai Rosai Hospital, Amagasaki, Japan (M.M., Y.M., H.U., H.O., S.K., M. Ochi, T. Mano, T.K.).; Department of Cardiovascular Medicine (A.S., T.O., H.K., T.D., Y. Sotomi, Y. Sakata) and Osaka University Graduate School of Medicine, Suita, Japan.; Cardiovascular Center, Sakurabashi Watanabe Advanced Healthcare Hospital, Osaka, Japan (N.T., M. Okada, K.T.).; Division of Cardiology, Osaka General Medical Center, Osaka, Japan (T.W., M.K.).; Cardiovascular Division, Osaka Keisatsu Hospital, Osaka, Japan (H.M., N.M.).; Division of Cardiology, Osaka Rosai Hospital, Sakai, Japan (Y.E.).; Department of Cardiovascular Medicine, Yao Municipal Hospital, Yao, Japan (T. Minamisaka).; Cardiovascular Medicine, Nara Medical University, Kashihara, Japan (S.H.).; Cardiovascular Division, National Hospital Organization, Osaka National Hospital, Osaka, Japan (K.I.).

**Keywords:** atrial fibrillation, atrial remodeling, pulmonary vein, recurrence, tachycardia, supraventricular

## Abstract

**BACKGROUND:**

In the randomized controlled SUPPRESS-AF trial (Efficacy and Safety of Left Atrial Low-voltage Area Guided Ablation for Recurrence Prevention Compared to Pulmonary Vein Isolation Alone in Patients with Persistent Atrial Fibrillation), the efficacy of low-voltage-area (LVA) ablation was highly dependent on the degree of atrial remodeling, while the efficacy was not statistically significant in total patients. This subanalysis of the SUPPRESS-AF trial aimed to compare the efficacy of LVA ablation in patient groups classified by left atrial diameter (LAD), which is a commonly used atrial remodeling index.

**METHODS:**

The SUPPRESS-AF trial included patients with persistent AF and left atrial LVAs, and compared rhythm outcomes between patients randomized to undergo pulmonary vein isolation (PVI) followed by left atrial LVA ablation group (n=170) or PVI-alone group (n=172). In this post hoc subanalysis, patients in each of the 2 randomly allocated groups were further divided into 2 groups using a median LAD of 44 mm.

**RESULTS:**

Atrial fibrillation or atrial tachycardia recurrence–free rates did not differ between patients with LAD>44 mm and ≤44 mm (60.1% versus 53.7%; *P*=0.261). Among patients with a LAD>44 mm, the LVA ablation group demonstrated a higher atrial fibrillation or atrial tachycardia-recurrence–free rate than the PVI-alone group (62.5% versus 43.4%; *P*=0.016). In contrast, no difference in atrial fibrillation or atrial tachycardia recurrence–free rate was found between the 2 groups of patients with a LAD≤44 mm (60.8% versus 59.6%; *P*=0.986).

**CONCLUSIONS:**

The efficacy of LVA ablation in addition to PVI for the treatment of persistent AF was more pronounced in patients with a large left atrium.

**REGISTRATION:**

URL: https://www.umin.ac.jp/ctr; Unique identifier: UMIN000035940.

**WHAT IS KNOWN?**

- SUPPRESS-AF trial demonstrated that low-voltage-area (LVA) ablation showed no significant benefit overall in persistent atrial fibrillation with left atrial LVAs.
- The trial suggested LVA ablation efficacy depends strongly on the degree of atrial remodeling.

**WHAT THE STUDY ADDS**

- In patients with left atrial diameter >44 mm, adding LVA ablation to pulmonary vein isolation improved atrial fibrillation or atrial tachycardia recurrence-free rates versus pulmonary vein isolation alone.
- The benefit of LVA ablation (versus pulmonary vein isolation alone) increased as left atrial size became larger.

Pulmonary vein isolation (PVI) is the cornerstone of ablation for persistent atrial fibrillation (AF), but its therapeutic effect is unsatisfactory.^1^ Low-voltage areas (LVAs) are thought to be associated with degenerated myocardium, including fibrosis,^2^ and possibly involve arrhythmogenic substrate serving as AF maintenance.^3,4^ Although several randomized controlled trials have evaluated the efficacy of ablation targeting LVAs, the efficacy of LVA ablation (LVA-ABL) was inconsistent.^5–8^ The randomized controlled SUPPRESS-AF trial (Efficacy and Safety of Left Atrial Low-voltage Area Guided Ablation for Recurrence Prevention Compared to Pulmonary Vein Isolation Alone in Patients with Persistent Atrial Fibrillation) showed that, although the efficacy of LVA-ABL was not statistically significant in total patients, the efficacy, nevertheless, appeared highly dependent on the degree of atrial remodeling.^9^

Left atrial enlargement is a well-known clinical feature of atrial remodeling. In patients undergoing AF ablation, left atrial enlargement has been repeatedly reported to be associated with poor rhythm outcomes.^10,11^ We, therefore, speculated that left atrial size may be used to identify a subset of patients who would benefit from LVA-ABL.

Here, we conducted a subanalysis of the SUPPRES-AF trial to investigate the efficacy of LVA-ABL in addition to PVI in subgroups classified by left atrial size.

## Methods

The data that support the findings of this study are available from the corresponding author on reasonable request.

### Study Design

This study is a post hoc analysis of the SUPPRESS-AF trial, an investigator-initiated, prospective, multicenter, randomized controlled trial which compared 1-year rhythm outcomes between 2 ablation strategies—PVI followed by left atrial LVA-ABL group and PVI-alone group—in patients with persistent AF and left atrial LVAs.^12,13^ LVA presence was defined when areas with a bipolar peak-to-peak voltage of <0.50 mV covered ≥5 cm^2^ of the left atrial surface. Patients in the SUPPRESS-AF trial were divided into 2 groups by median left atrial diameter (LAD). One-year rhythm outcomes were compared between randomized ablation strategies for each group with high and low LAD. All patients provided written informed consent to participate in the SUPPRESS-AF trial. The study conformed to the ethical guidelines outlined in the Declaration of Helsinki and was approved by the ethics committee of each participating hospital.

### Study Patients

**Figure 1.**
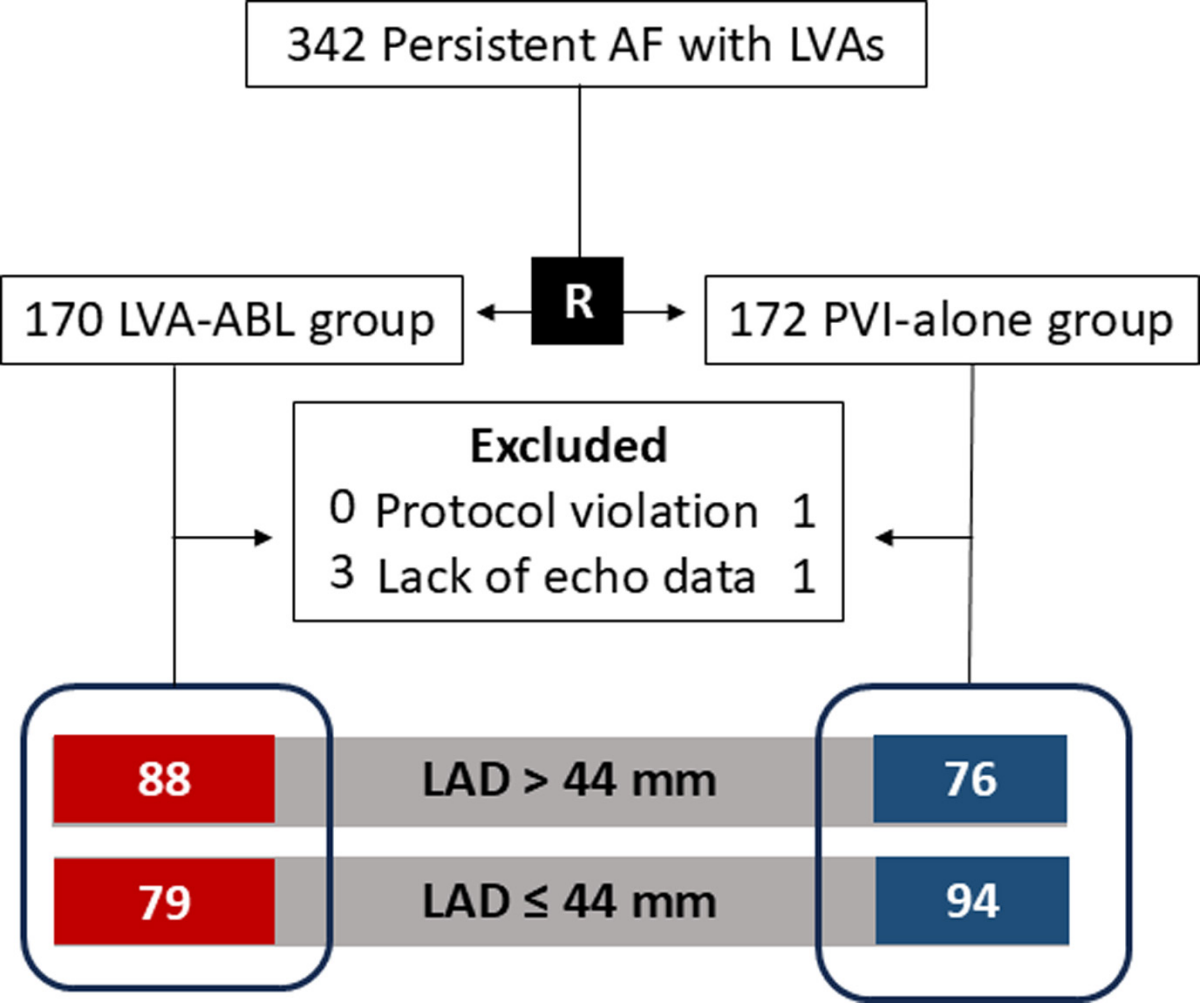
**Patient flow.** Among 342 patients enrolled in the SUPPRESS-AF trial, 337 patients were finally included in this study. All patients in each of the 2 randomly allocated groups were then further divided into 2 groups, those with a left atrial diameter (LAD) of >44 mm and ≤44 mm. AF indicates atrial fibrillation; LVA, low-voltage area; LVA-ABL, low-voltage-area ablation;PVI, pulmonary vein isolation; and SUPPRESS-AF, the Efficacy and Safety of Left Atrial Low-voltage Area Guided Ablation for Recurrence Prevention Compared to Pulmonary Vein Isolation Alone in Patients with Persistent Atrial Fibrillation trial.

The SUPPRESS-AF trial enrolled patients with persistent AF and left atrial LVAs. Key exclusion criteria were a LAD≥55 mm, history of cardiac surgery, valvular AF, hemodialysis, recent stroke, treatable cause of AF, and the physician’s judgment of unsuitability for enrollment. A total of 342 patients were enrolled and randomly assigned in a 1:1 ratio to either an LVA-ABL group (n=170) or a PVI-alone group (n=172, Figure 1). After exclusion of 1 patient for protocol violation and 4 patients for lack of preprocedural echocardiographic data, 337 patients were finally included.

Echocardiography was performed within 1 month before the ablation procedure. Anterior-posterior LAD was measured in the parasternal long-axis view using M-mode echocardiography. Using a median LAD of 44 mm, the patients in the either of the 2 randomly allocated groups were further divided into 2 groups with an LAD of >44 mm and ≤44 mm. Finally, the number of patients in the 4 groups was as follows: 79 in the LAD≤44 mm and LVA-ABL group; 94 in the LAD≤44 mm and PVI-alone group; 88 in the LAD>44 mm and LVA-ABL group; and 76 in the LAD>44 mm and PVI-alone group.

### Study Procedure

Ipsilateral encircling PVI was performed in all patients using an open-irrigated ablation catheter with a contact force sensor (Thermocool Smarttouch SF; Biosense Webster). Radiofrequency energy application was guided using a Visitag Surpoint module (Biosense Webster). Target Visitag Surpoint was set at ≥425 for the anterior wall and ≥375 for the posterior wall with an interlesion distance of ≤4 mm.

After the PVI, left atrial voltage mapping was performed under 100-ppm pacing from the high right atrium. Magnetic sensor-enabled multielectrode mapping catheters with a 1-mm electrode size (Lasso Nav or Pentaray, Biosense Webster) were used. Mapping points were automatically acquired using the Confidense Module (Biosense Webster) until filling all color gaps on the voltage map with fill and color interpolation threshold of 10 mm. LVAs were defined as areas with a bipolar peak-to-peak voltage of <0.5 mV. The scar level was set at 0.05 mV. The area of the LVA was manually measured using the area measurement tool of the CARTO system.

In the PVI+LVA-ABL group, homogenization ablation covering all LVAs was performed, except for scar areas where mapping points with voltage <0.05 mV concentrated. LVAs located at the posterior wall were allowed to be isolated with roof and bottom lines. Each radiofrequency application was guided by a Visitag Surpoint of ≥350 with an interlesion distance of <6 mm. It was acceptable to omit ablation at sites where ablation could impair physiological electrical conduction system or damage collateral structures, including the esophagus.

After completion of these procedures, induction of AF or atrial tachycardia (AF/AT) by atrial burst stimulation and intravenous administration of isoproterenol were performed. Induced AT and nonpulmonary vein AF triggers could be ablated at the discretion of the attending operator. In addition, cavo-tricuspid isthmus ablation was permitted when tricuspid isthmus-dependent atrial flutter was clinically observed.

### Follow-Up Protocol and End Points

Patients were followed for 12 months. A 12-lead ECG and 24-hour Holter ECG were acquired at 6 and 12 months. Twice-daily 30-s rhythm checks and symptom-driven checks were performed with a portable ECG (HCG 901 or HCG 801; Omron, Kyoto, Japan) from 6 to 12 months. AF/AT recurrence was defined as the occurrence of either of the following events: (1) AF/AT indicated on a scheduled or symptom-triggered ECG; or (2) AF/AT of at least 30-s duration on Holter ECG monitoring. AF/AT episodes during the 3 months after the initial or repeat ablation were not included as recurrence events (blanking period). Antiarrhythmic drug use was not recommended for 3 months after the ablation procedure. Primary end point was the recurrence of AF/AT without antiarrhythmic drug use during the 1-year follow-up period after the index ablation procedure.

### Statistical Analysis

Continuous variables are presented as the mean±SD or median (interquartile range), and categorical data as counts and percentages. Tests for significance were conducted using the unpaired *t* test or a nonparametric test (Mann-Whitney *U* test) for continuous variables, and the χ^2^ test or Fisher's exact test for categorical variables. To assess correlations between the continuous variables, Pearson correlation coefficient analysis was performed. Event-free rate was calculated using the Kaplan-Meier method. Survival curves between groups were compared using the 2-sided Mantel-Haenszel (log-rank) test. Hazard ratio, 95% CI, and *P* value for interaction were calculated with a Cox proportional hazards model. The proportional hazards assumption was tested by examining Schoenfeld residuals and by visual inspection of log-minus-log survival plots. The assumption was found to be adequately met. Impacts of LVA-ABL were shown using hazard ratios of the LVA-ABL group to the PVI-alone group. All analyses were performed using commercial software (SPSS version 26.0, SPSS, Inc, Chicago IL).

## Results

### Patient and Procedural Characteristics

Patient characteristics are presented in Table 1. Patients with LAD>44 mm were younger, had higher body mass index, higher systolic blood pressure, and longer AF duration than those with LAD≤44 mm. Concomitant diseases, including hypertension and heart failure, were more frequent in patients with LAD>44 mm. Echocardiographic data showed that patients with LAD>44 mm had more remodeling of the left ventricle, represented by a larger left ventricular diameter and lower ejection fraction.

**Table 1.**
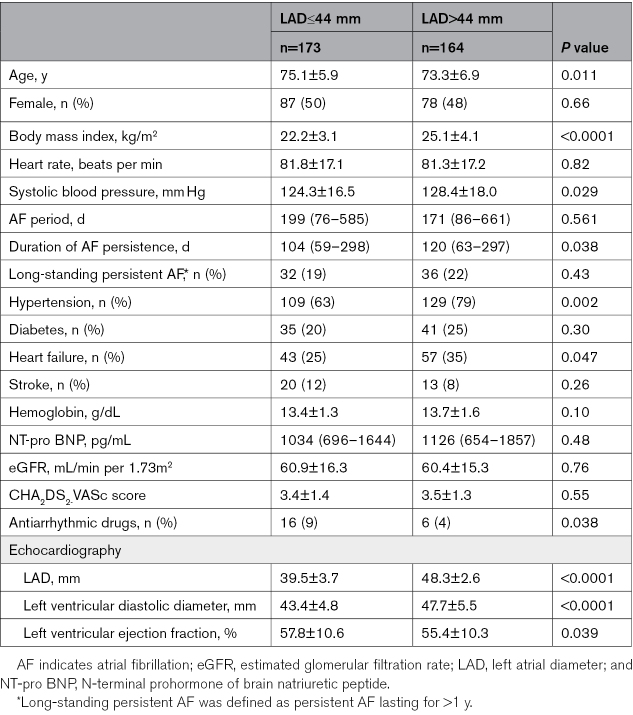
Baseline Characteristics

### Procedural Characteristics

The LVA-ABL group demonstrated a longer procedure time and longer radiofrequency-application time than the PVI-alone group, irrespective of left atrial size (Table 2). Ablation of regular AT was more frequently performed in the LVA-ABL group than the PVI-alone group, only in patients with an LAD <44 mm.

**Table 2.**
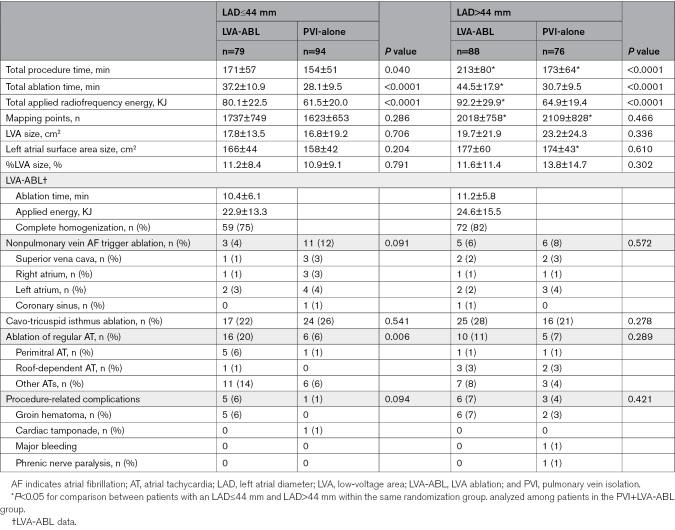
Procedural Characteristics

The number of mapping points and LVA size were greater in patients with an LAD>44 mm than LAD≤44 mm. However, almost no correlation was found between LAD and LVA size, with a correlation coefficient of 0.15 (*P*=0.006; Figure S1). In addition, procedural time and radiofrequency-application time among the LVA-ABL groups were longer in patients with LAD>44 mm than LAD≤44 mm. On the contrary, among the PVI-alone groups, these did not differ between groups by left atrial size.

### Efficacy of LVA-ABL in Relation to LAD

**Figure 2.**
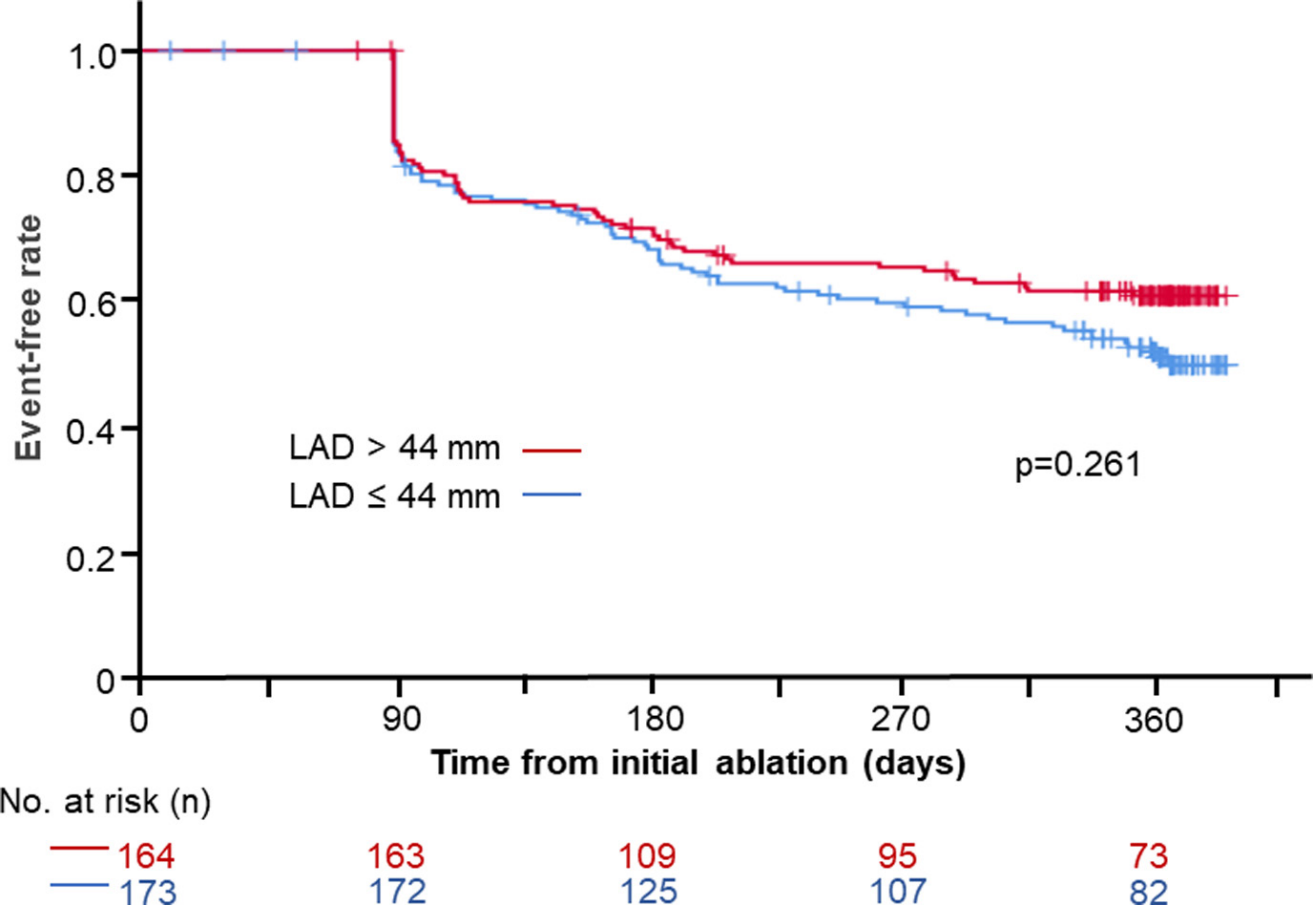
**Comparison of atrial fibrillation or atrial tachycardia (AF/AT) recurrence-free rate between groups classified by left atrial diameter (LAD).** AF/AT recurrence-free rates after the initial ablation without antiarrhythmic drug use. There was no difference in AF/AT recurrence rates between patients with an LAD>44 mm and ≤44 mm.

**Figure 3.**
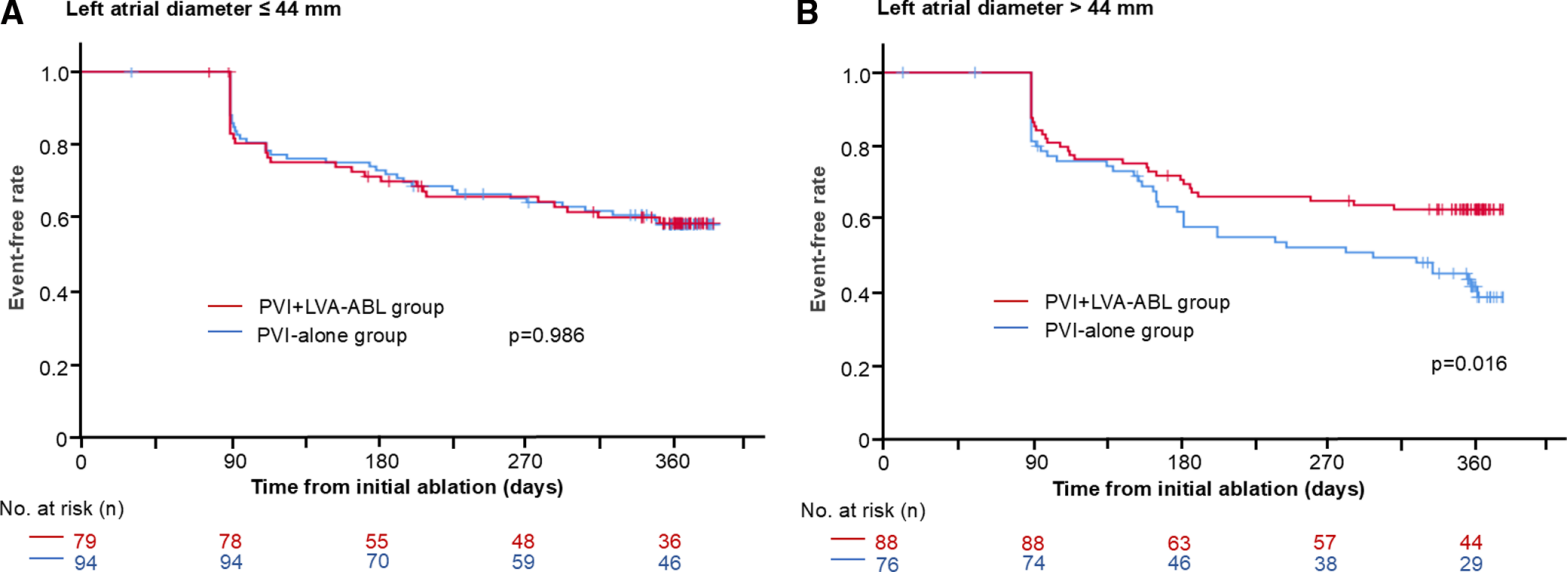
**Comparison of atrial fibrillation or atrial tachycardia (AF/AT) recurrence-free rate between the low-voltage-area ablation (LVA-ABL) group and pulmonary vein isolation (PVI)–alone strategy by patient groups classified by left atrial diameter (LAD).** AF/AT recurrence-free rates after the initial ablation without antiarrhythmic drug use. No difference in AF/AT recurrence rate was found between the LVA-ABL group and PVI-alone group in patients with an LAD≤44 mm (**A**). In contrast, among patients with an LAD>44 mm, the LVA-ABL group demonstrated a higher AF/AT recurrence-free rate than the PVI-alone group (**B**).

**Figure 4.**
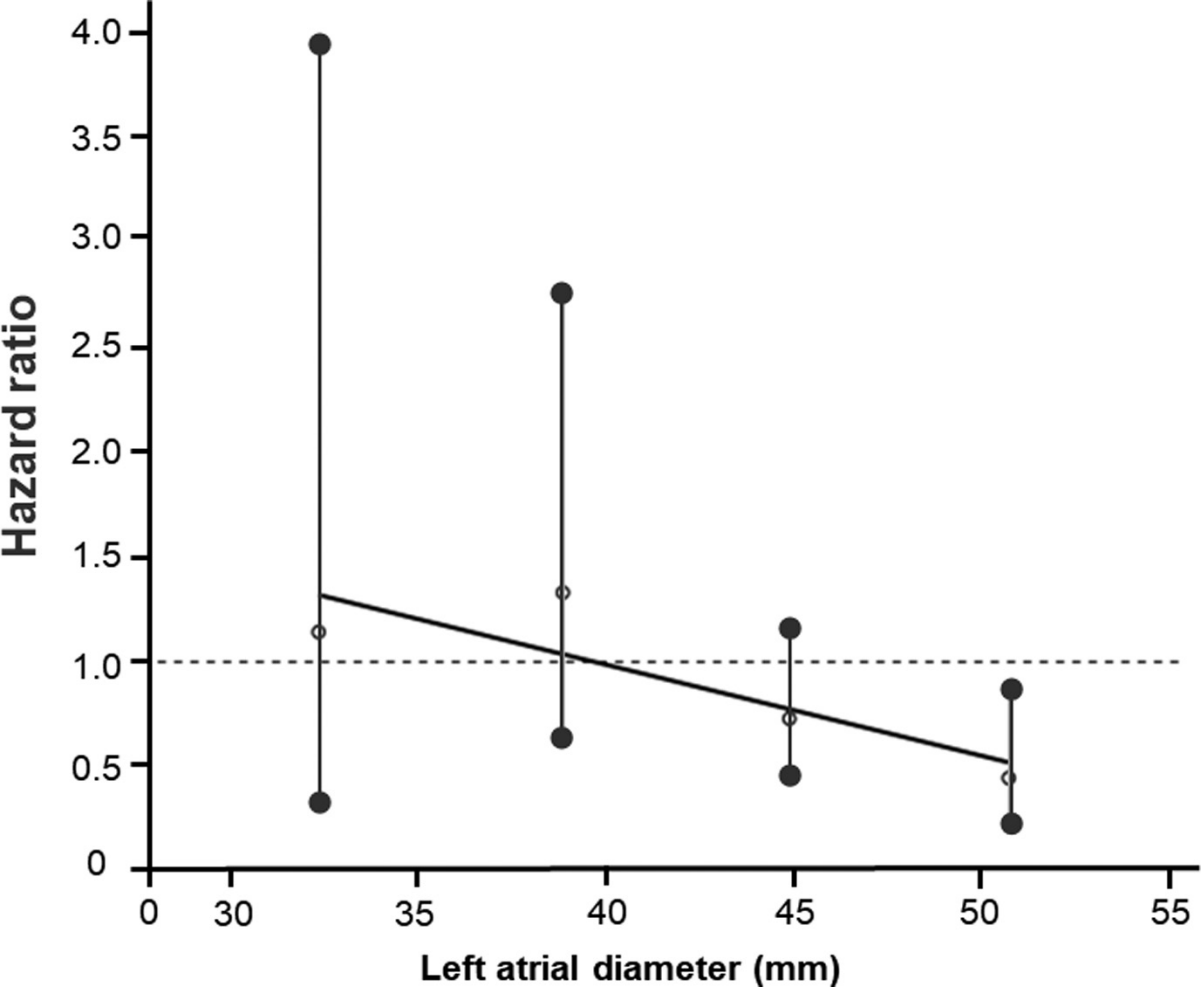
**Degree of left atrial enlargement and the benefit of low-voltage-area ablation (LVA-ABL).** Hazard ratios (open circles), 95% CIs (black circles), and approximate straight lines (black lines) of the primary end point in the LVA-ABL group to the pulmonary vein isolation–alone group stratified by left atrial diameter. Number of atrial fibrillation or atrial tachycardia recurrences and number of patients included in each left atrial size quartile were 10 events and 24 patients for 28.6 to 35.1 cm; 29 events and 83 patients for 35.1 to 41.7 cm; 71 events and 156 patients for 41.7 to 48.2 cm; and 36 events and 74 patients for 48.2 to 54.7 cm, respectively.

There was no difference in AF/AT recurrence rates between patients with LAD>44 mm and ≤44 mm (Figure 2). Among patients with LAD>44 mm, the LVA-ABL group demonstrated a higher AF/AT recurrence-free rate than the PVI-alone group (Figure 3A). On the other hand, no difference in AF/AT recurrence-free rate was found between the LVA-ABL group and PVI-alone group in patients with LAD≤44 mm (Figure 3B). Hazard ratios of AF/AT recurrence in the LVA-ABL group to PVI-alone group according to LAD are shown in Figure 4. The efficacy of LVA-ABL appeared to become more pronounced with increasing LAD.

**Figure 5.**
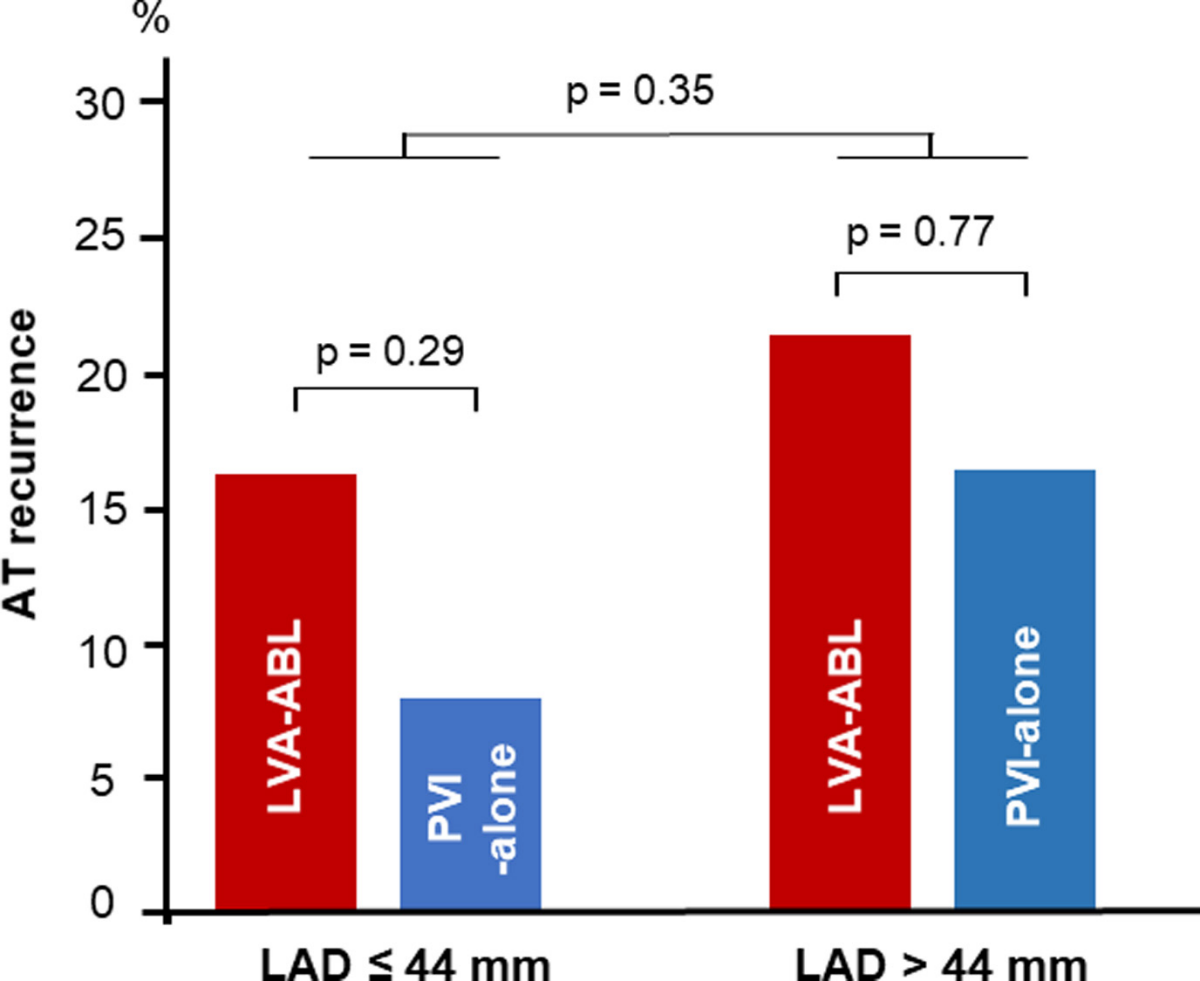
**Atrial tachycardia (AT) recurrence.** The proportion of ATs on the ECG for diagnosis of AF/AT recurrence. No statistically significant differences were found between groups in left atrial diameter (LAD) and ablation strategy. LVA-ABL indicates low-voltage-area ablation; and PVI, pulmonary vein isolation.

Among all patients with AF/AT recurrence, the proportion of ATs on the ECG for diagnosis of AF/AT recurrence is shown in Figure 5. No statistically significant differences between groups by LAD size and ablation strategy were found.

### Subgroup Analysis

The effect of LVA-ABL for each patient group classified by clinical background was examined in patients with an LAD>44 mm and ≤44 mm (Figures S2 and S3). In patients with LAD>44 mm, subgroups demonstrating the efficacy of LVA-ABL were female, age≥75 years old, body mass index <25.0 kg/m^2^, CHA_2_DS_2_VASc ≥4, left ventricular ejection fraction ≥50%, New York Heart Association functional class ≥II, AF persistence <1 year, concomitant hypertension, absence of diabetes, and LVA size ≥20 cm^2^. In patients with LAD≤44, individual subgroup analyses did not demonstrate a benefit of LVA-ABL, consistent with the results of the overall analysis.

## Discussion

The main findings of this post hoc analysis of the SUPPRESS-AF trial were as follows. (1) In patients with an LAD>44 mm, the LVA-ABL group demonstrated a higher AF/AT recurrence–free rate than the PVI-alone group. (2) In contrast, no difference in AF/AT recurrence rate was found between the 2 groups in patients with an LAD≤44 mm. (3) The hazard ratio of AF/AT recurrence in the LVA-ABL group to PVI-alone group decreased as the left atrium became larger. (4) Subgroup analysis suggested that the efficacy of LVA-ABL was associated with several clinical characteristics in patients with LAD>44 mm. In contrast, in patients with an LAD≤44 mm, no consistent efficacy of LVA-ABL was seen, irrespective of clinical characteristics.

### Pathophysiological Implication of Left Atrial Enlargement in the Formation of LVA

No or only a slight correlation was found between LVA size and LAD in this study. This finding is consistent with a previous study involving 1118 cases undergoing AF ablation.^12^ Of note, patient background in the present study was characterized by the degree of left atrial enlargement as follows: concomitant heart failure, hypertension, and long duration of AF persistence in patients with LAD>44 mm, and older and lower body mass index in patients with LAD≤44 mm. Similar clinical features dependent on left atrial size have been reported previously^12,13^ Taken together with the absence of correlation between left atrial size and LVA size and patient background variance by the degree of left atrial enlargement, differences in left atrial size may reflect different pathologies in the formation of fibrosis or other degeneration of the left atrium.

Cases with an enlarged left atrium are most commonly characterized by pressure and volume overload in the left atrium. Atrial stretch is reported to promote multiple factors, including atrial natriuretic peptide, angiotensin II, endothelin 1, transforming growth factor-beta, and inflammatory cytokines, leading to atrial enlargement.^14,15^ This theoretical explanation describes the atrial remodeling and tissue degeneration that accompany chamber enlargement.

On the other hand, patients with no or only mild left atrial enlargement were older and had lower body mass index. These factors result in atrial remodeling without any predominant involvement of an elevation of atrial pressure, and are therefore unlikely to be accompanied by atrial enlargement.^16,17^ In addition, atrial enlargement in these populations may be hindered by the diminished space available for atrial enlargement due to surrounding organs or structures.

### Impact of Left Atrial Enlargement on the Efficacy of LVA-ABL

We offer the following speculation on why LVA-ABL was effective only in patients with left atrial enlargement. First, left atrial size is a potentially good patient selection indicator for LVA-ABL, because the arrhythmic substrate is likely more prevalent in the atria outside the pulmonary veins in patients with advanced atrial enlargement. This concept is supported by a previous report, which demonstrated an association between left atrial enlargement and refractoriness to PVI in the presence of LVAs.^12^

Second, the difference in underlying pathology may impact the efficacy of LVA-ABL. In patients with advanced left atrial enlargement, suppression of AF by ablation leads to an improvement in conditions such as heart failure and AF persistence. Reduction of left atrial pressure and volume overload would, therefore, prevent further remodeling development, possibly contributing to a long-term AF suppression effect of LVA-ABL. On the other hand, atrial remodeling likely continues even after LVA-ABL in patients with no or mild left atrial enlargement, characterized by elderly and low body mass index. Further remodeling advancement could offset the effect of AF suppression by ablation.

Third, the AF-suppressing effect of LVA-ABL may have been counteracted by ATs after LVA-ABL in patients with no or mildly enlarged left atrium. This notion is based on our present finding that ablation targeting ATs at the initial ablation and ATs during the 1-year follow-up period in the LVA-ABL group were more frequently observed in patients with an LAD≤44 mm than in those with an LAD>44 mm. One possible explanation is that there is relatively less residual excitable myocardium after LVA-ABL in patients with no or mild left atrial enlargement, making ATs more likely to develop.^18^

### Limitations

This study has several limitations. First, the timing of echocardiography and the method used to measure LAD were not necessarily standardized among institutions, which may have led to interinstitutional bias. Second, the median LAD of 44 mm was used as the cutoff value for dividing the 2 groups, but this may not be correct from a pathophysiological perspective. Third, some patients from the original cohort were excluded due to the lack of echocardiographic data. Fourth, it has been proposed that LAD is not appropriate as a structural remodeling index of the left atrium.^19^ More precise markers—such as left atrial volume or fibrosis quantification using magnetic resonance imaging—may provide better characterization and improve the accuracy of the analysis. Fifth, patients with an LAD>55 mm were excluded from the SUPPRESS-AF study, and we are therefore unable to verify the efficacy of LVA-ABL in cases with very severe left atrial enlargement. Finally, the original study included only patients with LVA; therefore, the results of this subanalysis cannot be generalized to the entire population with persistent AF.

### Conclusions

The efficacy of LVA-ABL in addition to PVI for the treatment of persistent AF was more pronounced in patients with advanced left atrial enlargement.

## ARTICLE INFORMATION

### Acknowledgments

The authors thank clinical engineers Takashi Sumigawa, Naoya Kurata, Yusuke Ikada, Yoshitaka Kikuchi, Atsushi Shiono, and Hiroshi Kobayashi for their dedication in protocol creation and operation of ablation-related equipment according to the protocol; and the clinical research coordinators Nagisa Yoshioka, Satomi Kishimoto, Kyoko Tatsumi, and Yumi Yoshida for their excellent assistance in data collection, data management and secretarial administration.

### Sources of Funding

This work was supported by Biosense Webster Inc, through the investigator-initiated study program IIS 510.

### Disclosures

Dr Masuda has received honoraria from Medtronic and Daiichi Sankyo; Dr Minamiguchi has received honoraria from Medtronic and Abbott, as well as technical guidance fees from Philips and Cook Medical; Dr Inoue has received honoraria from Johnson & Johnson; Dr Sakata has received honoraria from Nippon Boehringer Ingelheim. All of these are unrelated to the present research. The other authors report no conflicts.

### Supplemental Material

Figures S1–S3

List of Investigators

## Supplementary Material


