# Supplementary material for: Efficacy of Low-Voltage-Area Ablation Is Enhanced in Patients With Advanced Left Atrial Enlargement: A Subanalysis of the SUPPRESS-AF Trial
Source: Circ Arrhythm Electrophysiol. 2025 Sep 26;18(10):e014210. doi: 10.1161/CIRCEP.125.014210 (PMC12529982; doi:10.1161/CIRCEP.125.014210)
Supplement: Supplementary file 1 [file hae-18-e014210-s001.pdf]

SUPPLEMENTAL MATERIAL

Supplemental Figures

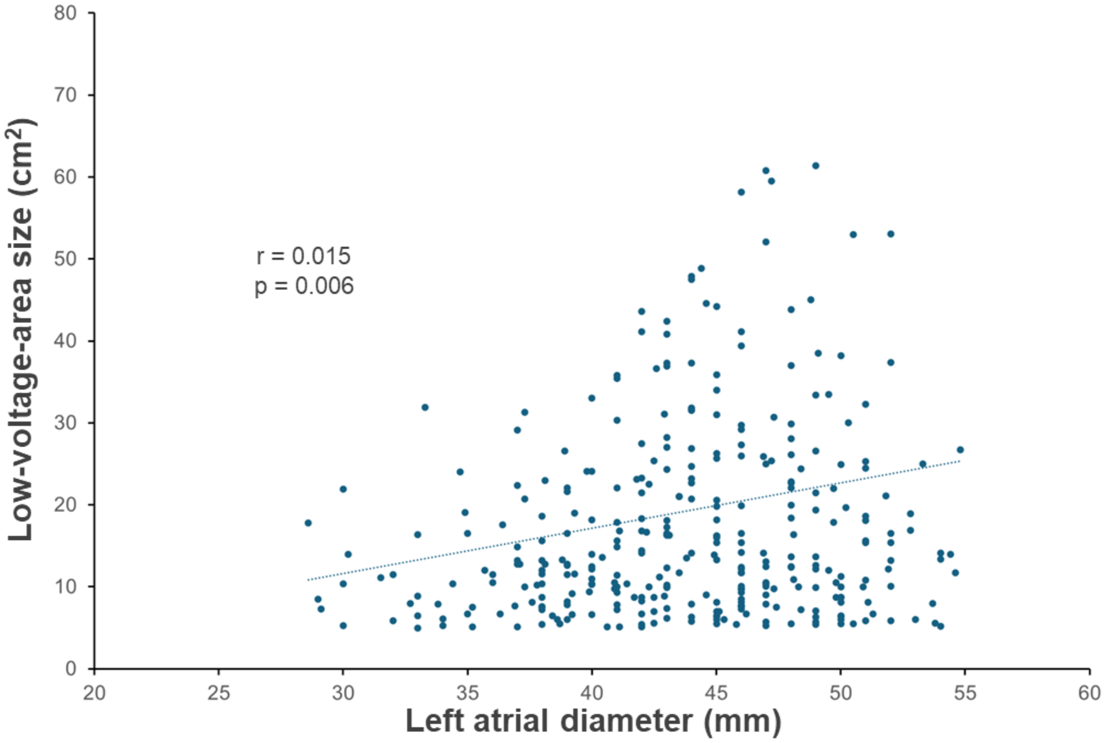

Supplementary Figure 1. Correlation between LAD and LVA size

LAD and LVA size plotted for each patient. Almost no correlation was found between LAD and LVA size. LAD, left atrial diameter; LVA, low-voltage area.

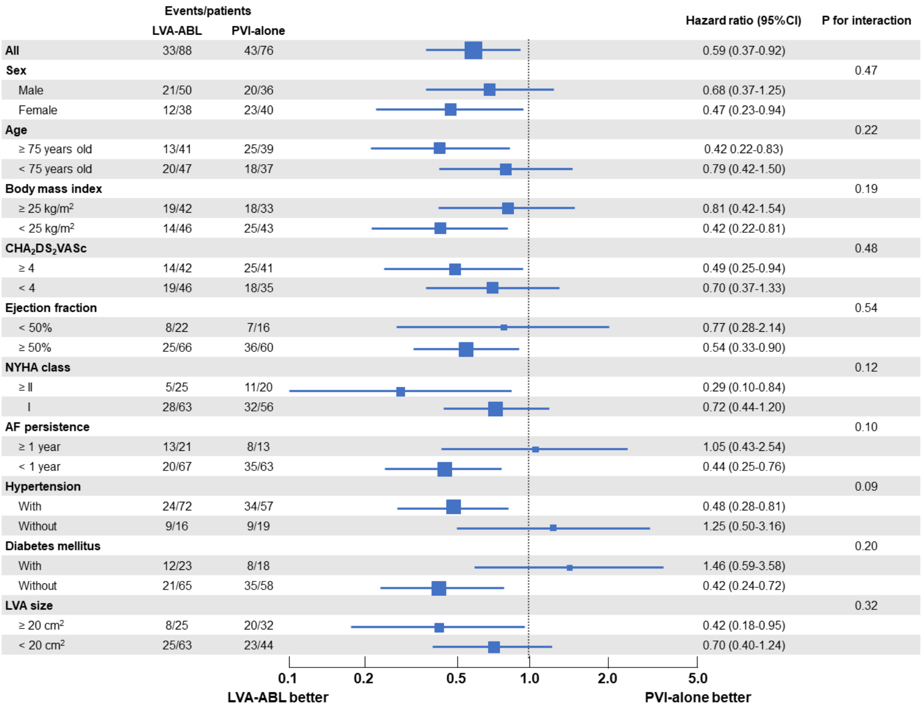

Supplementary Figure 2. Subgroup effects on AF/AT recurrence by randomized treatment strategy in patients with LAD > 44 mm

Forrest plots displaying hazard ratios and 95% CI of AF/AT recurrence in the LVA-ABL group compared with the PVI-alone group. CI, confidence interval; AF/AT, atrial fibrillation or atrial tachycardia.

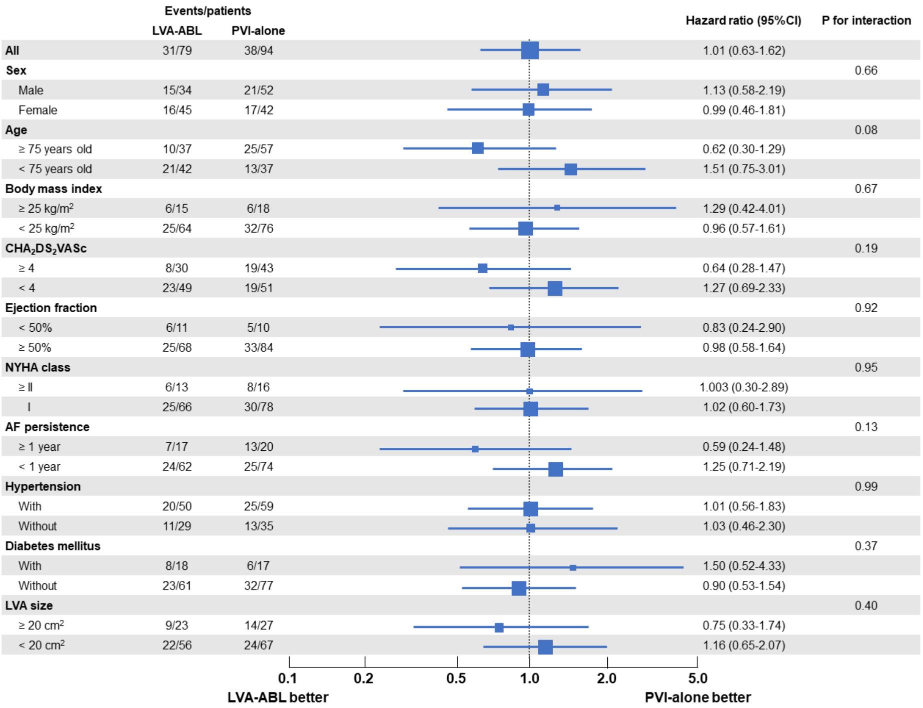

Supplementary Figure 3. Subgroup effects on AF/AT recurrence by randomized treatment strategy in patients with an LAD ≤ 44 mm

Forrest plots displaying hazard ratios and 95% CI of AF/AT recurrence in the LVA-ABL group compared with the PVI-alone group. CI, confidence interval; AF/AT, atrial fibrillation or atrial tachycardia.

List of Investigators

Steering Committee: Masaharu Masuda, Koichi Inoue, Tetsuya Watanabe, Nobuaki Tanaka, Hitoshi Minamiguchi, Yasuyuki Egami, Masato Kawasaki, Tomoko Minamisaka, Akihiro Sunaga, Tomoharu Dohi, Tomomi Yamada, Shungo Hikoso, Yohei Sotomi, Yasushi Sakata

Investigators:

Kansai Rosai Hospital, Amagasaki, Hyogo: Masaharu Masuda, Yasuhiro Matsuda, Hiroyuki Uematsu, Takuya Tsujimura, Hirotaka Ooka, Satoshi Kudo

Osaka University Graduate School of Medicine, Suita, Osaka: Akihiro Sunaga, Yuki Matsuoka, Daisaku Nakatani, Katsuki Okada, Hirota Kida, Daisuke Sakamoto, Tetsuhisa Kitamura, Takafumi Oka, Tomoaki Nakano, Takayuki Sekihara, Tomoharu Dohi, Tomomi Yamada, Shungo Hikoso, Yohei Sotomi, Yasushi Sakata,

Sakurabashi Watanabe Advanced Healthcare Hospital, Osaka, Osaka: Nobuaki Tanaka, Yuko Hirao, Koji Tanaka, Masato Okada, Kohei Iwasa

Yao Municipal Hospital, Yao, Osaka: Tomoko Minamisaka, Tetsuya Watanabe, Nobutaka Masunaga, Yukinori Sinoda, Norioki Inui, Ryohei Amiya, Masato Fujiwara, Arisa Murakami

Osaka Keisatsu Hospital, Osaka, Osaka: Hitoshi Minamiguchi, Yoshiharu Higuchi, Yasuhiro Ichibori, Naoki Mori, Takashi Kanda, Yuma Hamanaka, Yumi Tsutsui, Mikiko Matsumura, Takashige Sakio, Satoshi Nakawatase, Yuki Shibuya, Shinya Minami, Koichi Ochi, Ken Koda

Osaka Rosai Hospital, Sakai, Osaka: Yasuyuki Egami, Masamichi Yano, Yasuharu Matsunaga, Koji Yasumoto, Kohei Ukita, Mizuki Osuga, Noriyuki Kobayashi, Ayako Sugino

Osaka General Medical Center, Osaka, Osaka: Masato Kawasaki, Mitsutoshi Asai, Atsushi Kikuchi, Takumi Kondo, Tsutomu Kawai, Masahiro Seo, Atsushi Nakamura, Takeshi Fujita, Takuya Omoto, Yuki Kokubu, Yuto Fukuda

National Hospital Organization Osaka National Hospital, Osaka, Osaka: Koichi Inoue, Tuyoshi Mishima, Tatsuhisa Ozaki, Takuya Ohashi
